# The Role of Predictability During Negation Processing in Truth-Value Judgment Tasks

**DOI:** 10.1007/s10936-021-09804-0

**Published:** 2021-10-21

**Authors:** Franziska Rück, Carolin Dudschig, Ian G. Mackenzie, Anne Vogt, Hartmut Leuthold, Barbara Kaup

**Affiliations:** 1 Department of Psychology, University of Tübingen, Schleichstrasse 4, 72076 Tübingen, Germany; 2 Department of Psychology, Humboldt-Universität zu Berlin, Berlin, Germany

**Keywords:** Negation, Lexical association, Predictability, Sentence verification

## Abstract

In experiments investigating the processing of true and false negative sentences, it is often reported that polarity interacts with truth-value, in the sense that true sentences lead to faster reaction times than false sentences in affirmative conditions whereas the same does not hold for negative sentences. Various reasons for this difference between affirmative and negative sentences have been discussed in the literature (e.g., lexical associations, predictability, ease of comparing sentence and world). In the present study, we excluded lexical associations as a potential influencing factor. Participants saw artificial visual worlds (e.g., a white square and a black circle) and corresponding sentences (i.e., “The square/circle is (not) white”). The results showed a clear effect of truth-value for affirmative sentences (true faster than false) but not for negative sentences. This result implies that the well-known truth-value-by-polarity interaction cannot solely be due to long-term lexical associations. Additional predictability manipulations allowed us to also rule out an explanatory account that attributes the missing truth-value effect for negative sentences to low predictability. We also discuss the viability of an informativeness account.

## Introduction

When processing sentences, people often encounter difficulties. False sentences that convey information that is not true (e.g., “Zebras are dotted”) are especially difficult to process. We also see processing difficulties for sentences that contain a negation (e.g., Dudschig et al., 2018, 2019; Fischler et al., 1983; Kaup & Dudschig, 2020; Kaup et al., 2006). Interestingly, the two factors typically interact when it comes to processing difficulties. The pattern that emerges shows an advantage of true over false affirmatives, but curiously the same is not true for negative sentences. Here, true sentences (e.g., “Zebras are not dotted”) are either equally or even more difficult to process compared to false sentences (e.g., “Zebras are not stripy”).

There are several explanations why negated false sentences do not result in additive effects of negation and truth-value. Clark and Chase (1972) postulated a processing model for truth-value judgment in sentence-picture verification tasks. In their studies, participants viewed images (e.g., a star displayed above a cross) and indicated whether an affirmative (e.g., “The star is above the cross”) or negated sentence (e.g., “The cross is not above the star”) matches or mismatches the image. The model is based on the idea that participants represent the sentences and the images in an abstract propositional code (e.g., above[star, cross]) and then compare the two representations in a step-by-step fashion. More specifically, participants are assumed to compare each of the components of the two representations successively. Each time a mismatch is detected, they presumably change an internal response parameter (initialized as true) from true to false and vice versa. The status of this response parameter provides the result of the verification process, with the time required for responding being dependent on the number of times the response parameter has to be changed. The model nicely explains response time findings and, crucially, why there is no additive effect of the difficulties encountered in processing false and at the same time negative sentences. The verification of true affirmative sentences is easy because both representations already fully match (e.g., sentence: above[star, cross]; picture: above[star, cross]). False affirmative sentences are slightly more difficult as there is one mismatch (for instance, with respect to the order in which the objects appear in the brackets) and the response parameter has to be changed once (e.g., sentence: above[cross, star]; picture: above[star, cross]). For negative sentences, however, matters are reversed. False negative sentences only produce a mismatch in one component, namely the polarity operator (e.g., sentence: not[above[star, cross]]; picture: above[star, cross]) whereas true negative sentence produce a mismatch in two components, namely the polarity operator and the order in which the objects appear in the brackets (e.g., sentence: not[above[cross, star]]; picture: above[star, cross]). Thus, whereas false negative sentences require changing the response parameter only once, true negative sentences require changing it twice, making the latter more difficult to process. Applying this model to the question of why sentences such as “Zebras are not stripy” are rather quickly classified as false, one could argue that similar processes occur when participants compare sentences against their world knowledge (i.e. Zebras are stripy). Indeed, some years after the original model by Clark and Chase had been proposed, Carpenter and Just (1975) developed a similar model which however also covered verification processes involving world knowledge. Importantly, in the following years, these models were heavily criticized. For instance, Tanenhaus et al. (1976) pointed out that the representational component of the models describes highly task-specific “verification representations” (both for the sentences as well as the pictures and the background knowledge) but do not describe the processes by which these representations are derived. Thus, the corresponding models were taken to have a rather restricted scope, at best providing detailed descriptions of the verification processes taking place after participants have already understood the sentences.

Interestingly, however, in studies in which participants were not instructed to verify sentences, false negative sentences also led to faster response times than true negative sentences, indicating that the truth-value-by-polarity interaction is not dependent on verification. For instance, in a study by Kaup et al. (2005), participants were asked to indicate whether or not the two objects in a picture had been mentioned in a preceding sentence. In experimental sentences the correct answer was always “yes” but the sentence (e.g., “The star is/is not above the plus”) was either true or false with respect to the picture. Even though the task did not require participants to verify the sentences, a truth-value-by-polarity-interaction emerged. Also, when looking at electrophysiological data, false negative sentences are associated with smaller N400 amplitudes than true negative sentences (e.g., Dudschig et al., 2019; Fischler et al., 1983). Considering that the N400 is an event-related potential (ERP) component that is usually interpreted as reflecting processes integral to sentence comprehension—for example lexical semantic or compositional semantic processes in direct interaction with discourse or world-knowledge (e.g., Dudschig et al., 2016a, b a/b; Nieuwland & Van Berkum, 2006; Hagoort et al., 2004)—instead of later reasoning processes, these results also indicate that the truth-value-by-polarity interaction is probably not solely related to late verification processes. More specifically, Fischler and colleagues (1983) examined the processing of negation in the context of sentences that match or mismatch semantic knowledge. The response times to sentences like “A sparrow is/is not a bird” or “A sparrow is/is not a vehicle” showed that true affirmatives were verified faster than false affirmatives, but false negatives faster than true negatives. Additionally, false affirmatives as well as true negatives showed a more pronounced N400 than the true affirmatives and the false negatives. The authors concluded that these ERPs reflect a semantic match or mismatch (like in the well-known study by Kutas & Hillyard, 1980). However, more recent experiments found a similar ERP pattern for affirmative and negative sentences which matched or mismatched general world knowledge (e.g., “Zebras are not stripy”; Dudschig et al., 2019), indicating that the observed pattern is not specific to semantic violations but generalizes to other types of violations as well.

One could argue that the results of both types of ERP studies (involving semantic or world-knowledge violations) reflect lexical associations rather than processes at the sentence level after the negation has been integrated into sentence meaning. False negated sentences typically show a high lexical association between the noun (e.g., “zebra”) and the adjective (e.g., “stripy”). This could be the reason why false negative sentences are associated with smaller N400 amplitudes than their true counterparts for which this is not the case (e.g., “Zebras are not dotted”; for evidence that the N400 is sensitive to lexical associations, see e.g., Koivisto & Revonsuo, 2001; Rhodes & Donaldson, 2008). Therefore, these studies do not provide comprehensive insights into the processes at play during negation processing.

Further research argues that predictability plays a crucial role for the often-observed polarity- by-truth-value-interaction effect. This research is based on the view that negation is typically investigated in paradigms where its use is not pragmatically licensed. Nieuwland and Kuperberg (2008) conducted an ERP study where negation use was licensed by the preceding context (e.g., “With proper equipment, scuba diving is not dangerous”). As predicted by the authors, true negative sentences in these contexts did not lead to increased processing costs as indicated by N400 amplitudes, eliminating the relative processing ease of false negated sentences (see also Dale & Duran, 2011, for similar results for mouse movement trajectories). Nieuwland (2016) followed up on this study and examined N400 amplitudes in sentences that varied in truth-value and polarity and that were controlled for cloze probability of the target words. The typical truth-value-by-polarity interaction was only observed for low cloze value items. For high cloze (and therefore predictable) items, the effect of truth-value was the same for affirmative and negative sentences. In other words, the relative processing ease for false negative sentences only showed in circumstances not allowing predictions. Taken together, current evidence from the ERP literature suggests that the often-observed truth-value-by-polarity interaction might actually be restricted to certain specific uses of negation, in particular to negation use in vague contexts that do not pragmatically license negation and do not allow the comprehender to predict upcoming linguistic material. We compiled an overview of the most relevant studies in Table 1 to show differences in the employed material regarding lexical associations and manipulation of predictability and how the current study contributes to the research.

**Table 1:**
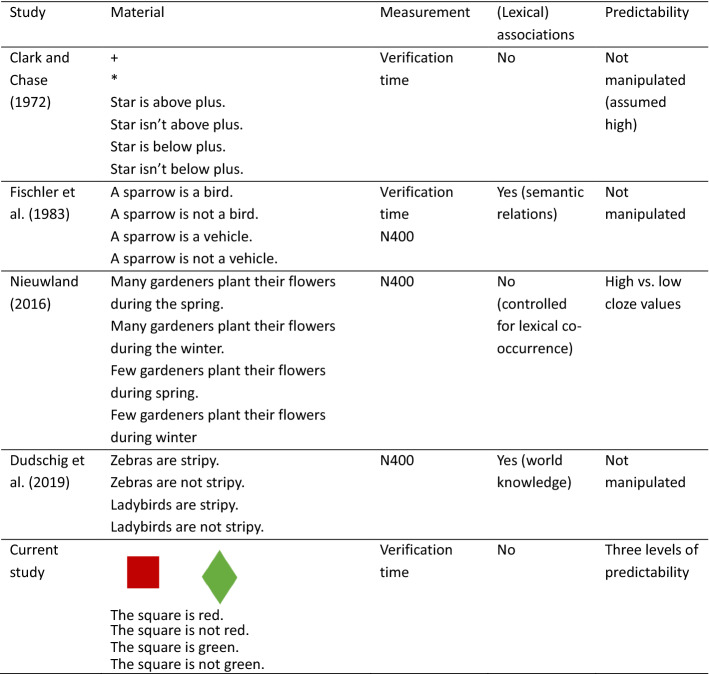
Overview of relevant studies and how they differ in material, measurement, lexical associations and manipulation of predictability

However, we would like to point out that the N400 research using negation needs to be interpreted carefully. On the one hand, some studies investigate the sensitivity of the N400 to negation, assuming that negation is instantly integrated during comprehension and ask whether the N400 reflects this integration (Nieuwland, 2016; Nieuwland & Kuperberg, 2008; Schiller et al., 2017). Other studies use the N400 as an indicator to investigate whether negation is indeed instantly integrated during processing (Dudschig et al., 2019; Fischler et al., 1983; Haase et al., 2019; Lüdtke et al., 2008; Palaz et al., 2020; Wiswede et al., 2013). The latter presupposes that the N400 would reflect such an integration if it had taken place. Thus, overall, the literature sometimes suffers from circularity in this domain. In our view, it is not yet clear how to comprehensively interpret all the N400 results in a converging direction regarding the mechanisms at play during negation processing. Therefore, the present study investigates the role of predictability in a behavioral paradigm that can be directly compared to early behavioral findings in the negation literature.

The aim of the present study was to use a behavioral paradigm that does not have issues regarding long-term lexical associations between the noun and predicate (e.g., “Zebras are stripy/dotted”), but nevertheless allows manipulating predictability. We constructed small artificial worlds using geometric shapes with particular colors, sizes, patterns, brightness (e.g., a large white square and a small or large blue circle) and presented them to participants together with true and false affirmative and negative sentences (e.g., “The circle is/is not white”). See Table 3 and the Appendix for examples. We reasoned that this would allow us to investigate whether false negatives are easy to process when there are no long-term lexical associations triggering this effect, and whether predictability has an influence on this processing advantage. All of our visual worlds consisted of two geometrical forms (e.g., a square and a circle) which could be unambiguously referred to. Apart from shape, the geometrical forms differed in one or two properties of color, pattern, brightness and/or size. Geometrical forms should typically not be lexically associated with any of these properties. A square should not be more associated with the color blue than a circle for most people (for exceptions, see Albertazzi et al., 2013). When the two forms of different shape only differ in one additional aspect (e.g., a blue circle and a red square of equal size, pattern, brightness), then the predictability of the last word of the sentence used to refer to one of the forms should be high, because there is only one dimension the sentence in this particular restricted context can refer to (e.g., “The circle is not red” or “The circle is blue”). When the forms differ in two additional aspects (e.g., color and pattern as in a world with a striped blue circle and a dotted white square), then the predictability of what aspect will be used to describe the form is lower (e.g., “The circle is not dotted” or “The circle is not white”). If lexical associations play an important role for the interaction between polarity and truth-value, our study should only produce a main effect of both polarity and truth-value, as negative sentences are usually harder to process than affirmatives and false sentences are harder than true sentences. The main effect of polarity is expected both for response times and error rates (faster and less error-prone responses in the affirmative compared to the negative conditions). Polarity should not interact with truth-value because in our sentences because there are no lexical associations between the adjectives and the nouns in any of the conditions. In contrast, if predictability is the key feature, we should see a facilitation for a true negative sentence such as “The circle is not white” in the high-predictable context compared to the low-predictable context, or in other words, we should see a truth-value-by-polarity interaction specifically in the low-predictable contexts but not in the high-predictable contexts. Again, this pattern should be reflected in both response times and error rates. For selecting our materials, we conducted a pretest. This pretest will be described in what follows before we turn to the actual experiment.

## Pre-test of the Material: Cloze Study

### Participants

We recruited 82 participants (44 male, 38 female) in the US through the Amazon’s Mechanical Turk (mTurk) platform to take part in a cloze pretest of the item material. The online study was programmed in jsPsych (de Leeuw, 2015) and lasted approximately 30 min. Participants received 6 US$ in return for their participation. We excluded one participant who did not answer according to the instructions but provided whole sentences, and one participant who did not list English as their native language. The age ranged from 22 to 71 years (*M* = 36.88, *SD* = 10.44). One participant did not list his or her age. The University’s Faculty of Science Ethics Committee for Psychological Research granted ethical approval for the experiment.

### Materials, Procedure and Item Selection

We used nine geometrical forms (arrow, circle, cross, diamond, oval, rectangle, square, star, triangle) with a different color (blue, green, orange, red, white), pattern (checkered, crosshatched, dotted, solid, striped), size (large, small) or brightness (light, dark). From these forms, we created 180 pairs of geometrical forms. For high-predictable pairs, the forms differed in only one aspect apart from their shape (e.g., in color, a blue circle and a red square), for low-predictable pairs, the forms differed in two aspects apart from their shape (e.g., color and size, a small blue circle and a big red square). There were 90 high- and 90 low-predictable pairs. Each pair was combined with an affirmative or negated sentence fragment that referred to either the left or the right form to yield a picture-fragment combination. The fragments were created by truncating the end of sentences that truly described the pictures. Affirmative and negative fragments were of the form “The form is” or “The form is not”, respectively. Each pair of geometrical forms was thus part of four picture-fragment combinations. The overall 720 possible picture-fragment combinations were divided into four lists of 180 trials. Each participant saw one list. The picture-fragment combinations were presented on a light grey background. Participants were instructed that the forms would differ in size, fill, color and brightness and they should complete the sentences with an adjective referring to one of these dimensions. They should further not use diminutive or comparative forms of adjectives or tautological completions (e.g., “The circle is round”). In each trial, participants first saw the two forms. After clicking a ‘Continue’ button, the truncated sentence appeared below the geometrical forms and participants completed it by typing in the sentence end.

We spellchecked the answers manually and corrected typos. Trials in which participants had not produced exactly one adjective or an answer that did not resemble the anticipated answers were counted as mistakes. Adjectives that were similar in meaning to the adjectives we had had in mind when creating the items were counted as correct (e.g., when the form was dotted, we also considered “spotted” or “polka dotted” as correct answers). Next, we excluded all those picture-fragment combinations that had less than 70% overall correct answers. From the remaining, we selected 40 picture-fragment combinations that were highly predictable, 20 of which with an affirmative and 20 with a negative sentence fragment. For the low-predictable pairs (in which the geometrical forms differed in two dimensions), we identified two varieties. We considered pairs as members of the “low_1_”-category when the majority of answers referred to one dimension (at least 70%) but none or very few answers referred to the other dimension (at most 25%). Pairs for which people referred to both dimensions approximately equally often (at most 50% each) were considered members of the “low_2_”-category. For each of the two types of low-predictable picture-fragment combinations, we identified 40, of which 20 were affirmative and 20 negative. Together with the 20 affirmative and 20 negative high-predictable picture-fragment combinations this resulted in a total of 120 combinations that could be used as stimuli in our main experiment. See Table 2 for the mean cloze values for the adjectives employed in the 12 conditions.

**Table 2:** Means and standard deviation for the cloze values (%) of the last word of the sentences

Sentence polarity and truth-value	Display version		
	High-predictable	Low_1_-predictable	Low_2_-predictable
Affirmative, true	88.50 (3.91)	3.25 (3.96)	31.50 (7.10)
Affirmative, false	1.75 (2.86)	0.25 (1.09)	0.75 (2.39)
Negative, true	83.50 (3.20)	9.25 (7.30)	32.50 (5.37)
Negative, false	3.00 (4.00)	0.50 (1.50)	1.50 (2.29)

It should be noted that due to the rather complicated selection procedures based on the close values determined in this pretest, the three levels of predictability were not matched with regard to lexical variables such as length and lexical frequency. More specifically, with regard to number of characters, the high-predictable category (*M* = 4.46, *SD* = 0.97) significantly differed from the low_1_ category (*M* = 5.12, *SD* = 1.34; *t*(143.5) = − 3.58, *p* < 0.001) and from the low_2_ category (*M* = 5.57, *SD* = 1.86; *t*(118.77) = − 4.74, *p* < 0.001); the two low-predictable categories did not differ significantly (*t*(143.80) = − 1.75, *p* = 0.082). Also, with regard to the frequency class, the predictability categories differed. Adjectives in the low_2_ category (*M* = 11.21, *SD* = 3.10) were less frequent than adjectives in the low_1_ category (*M* = 9.40, *SD* = 2.60; *t*(153.33) = 4.00, *p* < 0.001) and less frequent than adjectives in the high-predictable category (*M* = 9.87, *SD* = 0.97; *t*(94.36) = 3.68, *p* < 0.001). There was no difference between the high and low_1_ category (*t*(100.65) = 1.53, *p* = 0.129). Therefore, we conducted extra analyses with data sets matched with regard to either length or frequency of the adjectives for the three levels of predictability (see results section). However, it should also be noted that matching with respect to lexical variables is not strictly necessary in our case. Our manipulation of predictability did not aim at testing a main effect of predictability which would be easily explained by a confound in frequency or length. Rather it aimed at testing an account that predicts a three-way interaction between polarity, truth-value and predictability in the sense that the polarity-by-truth-value interaction is stronger for low-predictable cases.

As another side effect of the item selection procedure, items referring to the size of a shape were not evenly distributed across experimental conditions. Note, that to judge the truth-value of a size-related statement (e.g. “The circle is not big”), one would need to consider both shapes—contrary to a color- or fill-related statement (e.g. “The circle is not red”). This comparison could lead to longer verification times that, due to the uneven distribution of size-related statements, differentially contribute to performance in the different conditions. However, this should not mitigate the expected truth-value-by-polarity interaction, as also in the study of Clark and Chase (1972), both objects had to be taken into account when judging the truth-value of a sentence like “The star is not above the plus”.

Our material also included 31 items in which one form consisted of an arrow. Note, that arrows are different from other stimuli (e.g., squares) with regard to their spatial-directional nature, which in turn may impact on how these stimuli are processed. Specifically, arrows are known to elicit attentional shifts towards the indicated direction and have also been shown to prime spatially compatible responses in choice reaction-time tasks (e.g., Eimer, 1995). We addressed this issue by re-analyzing the data containing no arrow trials. See the Appendix 2 for the results.

## Main Experiment

### Participants

We recruited a new set of 50 English native speakers in the US through the mTurk platform. They stated that they had no visual impairments regarding the recognition of colors or patterns, like red-green visual impairment or color blindness. The online study was programmed in jsPsych (de Leeuw, 2015) and lasted approximately 30 min. Participants received 6 US$ in return for their participation. We excluded the data sets of 10 participants. These participants either answered by only pressing one response key throughout the whole experiment (N = 3), pressing the response key too early in more than 50% of the trials (N = 2), had an accuracy rate below 80% (N = 3) or did not list English as their native language (N = 2). The age of the 40 remaining participants ranged from 21 to 66 years (*M* = 37.35, *SD* = 10.48). Twenty-one were male. One participant did not list his or her age. The University’s Faculty of Science Ethics Committee for Psychological Research granted ethical approval for the experiment.

### Materials

Materials consisted of 120 picture-sentence combinations that were created on the basis of the 120 picture-fragment combinations selected in the pretest. More specifically, for each of the 20 combinations in the 3(type: high vs. low_1_ vs. low_2_) × 2(polarity) conditions, 10 were combined with a true and the remaining 10 were combined with a false sentence. For true sentences, we used the correct adjectives from the respective condition in the pre-test. In other words, for high-predictable-combinations we used the adjectives produced by the majority of the participants, for low_1_-combinations we used the adjectives produced by none or only a few of the participants and for low_2_-combinations we selected items with the second highest cloze probability or randomly, if probabilities were equally high. For false sentences, we replaced the adjective with the adjective that would be true when referring to the distractor object describing the same dimension. Thus, for a pair consisting of a blue circle and a red square, the sentence “The square is red” functioned as a true sentence, and the sentence “The square is blue” functioned as a false sentence (high-predictable). For a pair consisting of a blue circle and a checkered red circle, the sentence “The square is checkered” functioned as a true sentence, if less than 25% of the participants had used this adjective for this pair in the pre-test, and the sentence “The square is solid” functioned as the false sentence (low_1_-predictable). Finally, for a pair falling in the low_2_-category (e.g., consisting of a small blue circle and a large red square), we chose the true sentence to be “The square is large” if “large” was the second most frequent completion after “red” (“red” chosen in at most 50% of the cases), whereas “The square is small” served as a false sentence in this condition (low_2_-predictable). We included the two sets of low-predictable items to address the different patterns in the cloze completion pre-test. See Table 3 for an example of each category. Further examples of the material set are provided in the “Appendix”.

**Table 3:**
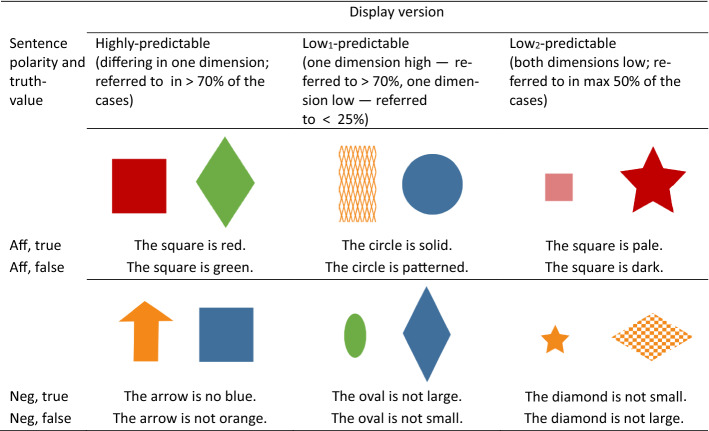
Examples for artificial visual worlds and true and false affirmative and negated sentences in the three predictability conditions

### Design and Procedure

We presented every participant with 120 picture-sentence combinations that were either affirmative or negative, high-, low_1_- or low_2_-predictable, and either true or false. Truth-value varied within items, i.e. for every true picture-sentence-combination, there was a false picture-sentence-combination, describing the same geometrical form with a non-matching adjective. Due to the item selection process, polarity and predictability varied between items, i.e. there were different picture-sentence-combinations for affirmative and negative items, as well as for the different levels of predictability. We created two sets of picture-sentence combinations so that each set included each picture only once. In each set, half of the combinations were true and half were false. Also, half were affirmative and half were negative. Finally, one-third of the combinations in each set were high-predictable, one-third were low_1_-predictable and one-third were low_2_-predictable.

A trial started with the presentation of a fixation cross for 400 ms. Then, the two geometrical forms were presented and 1000 ms later the sentence appeared underneath. Both sentence and geometrical forms stayed on screen until the participant’s keypress response. Participants had to indicate as quickly as possible whether the sentence was true or false regarding the presented picture by pressing the according keys on the keyboard. The keys C and M served as response keys. Participants were instructed to keep their left index finger on C and their right index finger on M. The mapping of the keys to the true vs. false response was made clear in the instructions. This mapping was counterbalanced between participants. Each participant saw one of the two sets of picture-sentence combinations. Together with the two response-key mappings, this resulted in four experimental lists. The order in which the individual picture-sentence-combinations were presented was random for each participant. Before the experimental session started, each participant completed four practice trials.

### Data Processing and Analyses

We analyzed response times and accuracy of responses. For analyses of response times, we excluded all trials with an incorrect answer. We also excluded outliers in two steps. First, we excluded all trials with response times shorter than 200 ms and longer than 7000 ms. Second, we z-transformed the response time data for every participant in every item condition and only included response times with corresponding z-values between -2 and 2. With this outlier correction, 5% of the correctly answered experimental trials were removed from the subsequent analyses.

### Results

**Fig. 1:**
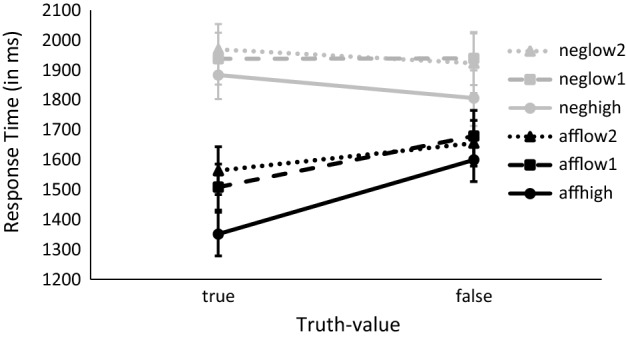
Response times for true and false sentences, separated for different combinations of polarity (aff = affirmative, neg = negated) and predictability (high = one differing property, true adjectives high-predictable, low_1_ = two properties that differ in predictability, true adjective low-predictable, low_2_ = two low-predictable properties, true adjective low-predictable). Errorbars show the standard error of the mean

We conducted two Huynh–Feldt corrected 2 × 2 × 3 ANOVAs with the factors polarity (affirmative, negated), truth-value (true, false) and predictability (high, low_1_, low_2_) on response times. One of these ANOVASs treated participants as random factor, the other treated items as random factor. Responses were slower for negated compared to affirmative statements (*F*_1_(1, 39) = 148.30, *p* < 0.001, η_G_^2^ = 0.10; *F*_2_(1, 114) = 175.22, *p* < 0.001, η_G_^2^ = 0.40), as well as for false compared to true statements (*F*_1_(1, 39) = 7.02, *p* = 0.011, η_G_^2^ = 0.004; *F*_2_(1, 114) = 4.04, *p* = 0.005, η_G_^2^ = 0.02). Response times also differed for the three levels of predictability (*F*_1_(2, 78) = 18.40, *p* < 0.001, η_G_^2^ = 0.01; *F*_2_(2, 114) = 7.96, *p* < 0.001, η_G_^2^ = 0.06), reflecting the advantage of high-predictable statements compared to statements in the category low_1_ (*t*_1_(317.67) = − 1.72, *p* = 0.086; *t*_2_(77.97) = -2.05, *p* = 0.043) and low_2_ (*t*_1_(317.56) = -1.90, *p* = 0.058; *t*_2_(77.63) = -2.33, *p* = 0.022) and no difference between the two low categories (*t*_1_(317.99) = -0.18, *p* = 0.853, *t*_2_(77.81) = -0.32, *p* = 0.746). Predictability did not interact with truth-value (*F*_1_(2, 78) = 1.04, *p* = . 349, ε = 0.84, η_G_^2^ = 0.001) nor with polarity (both *F*s < 1). There was however an interaction between polarity and truth-value (*F*_1_(1, 39) = 15.27*, p* < 0.001, η_G_^2^ = 0.01; *F*_2_(1, 114) = 13.90*, p* < 0.001, η_G_^2^ = 0.06). This pattern reflects the typical interaction between truth-value and polarity—particularly fast responses to true affirmative sentences compared to false affirmative as well as true and false negated sentences. There was no influence of predictability on the truth-value-by-polarity interaction (*F*_1_(2, 78) = 1.73,* p* = 0.182, η_G_^2^ = 0.001; *F*_2_(2, 114) = 1.38,* p* = 0.255, η_G_^2^ = 0.01). Separate analyses for the three levels of predictability revealed the interaction between truth-value and polarity only in high-predictable combinations (*F*_1_(1, 39) = 11.98, *p* = 0.001, η_G_^2^ = 0.03; *F*_2_(1, 38) = 12.96, *p* < 0.001, η_G_^2^ = 0.18), with faster responses to true affirmatives compared to false affirmatives (*t*_1_(39) = − 5.27, *p* < 0.001; *t*_2_(19) = − 3.96, *p* < 0.001), and no significant differences in response times for true negatives compared to false negatives (*t*_1_(39) = 1.21, *p* = 0.233; *t*_2_(19) = 1.34, *p* = 197). However, we did not see the interaction between truth-value and polarity in low-predictable combinations, at least not in both the by-participant as well as the by-item analyses (low_1_: *F*_1_(1, 39) = 7.56,* p* = 0.008, η_G_^2^ = 0.01; *F*_2_(1, 38) = 2.46,* p* = 0.125, η_G_^2^ = 0.04; low_2_: *F*_1_(1, 39) = 2.49,* p* = 0.122, η_G_^2^ = 0.004; *F*_2_(1, 38) = 1.82,* p* = 0.186, η_G_^2^ = 0.02). In the low_1_ condition, negative statements took longer to verify compared to affirmative statements (*F*_1_(1, 39) = 66.41, *p* < 0.001, η_G_^2^ = 0.10; *F*_2_(1, 38) = 63.34, *p* < 0.001, η_G_^2^ = 0.41) and false statements tended to take longer to verify than true statements (*F*_1_(1, 39) = 3.90, *p* = 0.055, η_G_^2^ = 0.01; *F*_2_(1, 38) = 3.06, *p* = 0.088, η_G_^2^ = 0.04). The relative ease to classify affirmative sentences also showed in the low_2_ condition (*F*_1_(1, 39) = 64.06, *p* < 0.001, η_G_^2^ = 0.09; *F*_2_(1, 38) = 36.69, *p* < 0.001, η_G_^2^ = 0.33). However, there was no difference in response times between true and false sentences (both *F*s < 1). The truth-value by polarity interaction in high-predictable combinations reflects again the typical pattern of relatively fast responses to true affirmative sentences rather than the hypothesized facilitation for true negated sentences in these contexts.^1^ See Fig. 1 for the response times for true and false sentences in the different conditions of polarity and predictability.

**Fig. 2:**
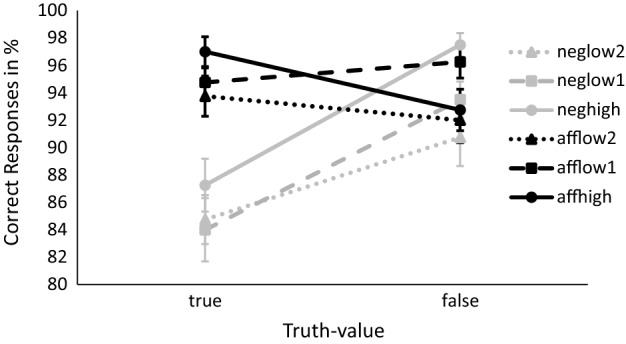
Percentage of correctly verified true and false sentences, separated for different combinations of polarity (aff = affirmative, neg = negated) and predictability (high = one differing property, true adjectives high-predictable, low_1_ = two properties that differ in predictability, true adjective low-predictable, low_2_ = two low-predictable properties, true adjective low-predictable). Errorbars indicate the standard error of the mean

We further tested the influence of polarity, truth-value and predictability on the accuracy of responses. The two Huynh–Feldt corrected 2 × 3 × 2 ANOVAs revealed a lower accuracy rate for negated statements compared to affirmatives (*F*_1_(1, 39) = 23.69, *p* < 0.001, η_G_^2^ = 0.05; *F*_2_(1, 114) = 30.17, *p* < 0.001, η_G_^2^ = 0.13), as well as for true compared to false statements (*F*_1_(1, 39) = 15.64, *p* < 0.001, η_G_^2^ = 0.03; *F*_2_(1, 114) = 22.63, *p* < 0.001, η_G_^2^ = 0.08). There was a main effect of predictability (*F*_1_(2, 78) = 6.20, *p* = 0.003, η_G_^2^ = 0.02; *F*_2_(2, 114) = 4.82, *p* < 0.001, η_G_^2^ = 0.05), reflecting a higher accuracy rate in the high condition compared to the low_2_ condition(*t*_1_(309.06) = 2.77, *p* = 0.006; *t*_2_(61.59) = 2.88, *p* = 0.005), but not the low_1_ condition (*t*_1_(314.47) = 1.32, *p* = 0.194; *t*_2_(64.82) = 1.39, *p* = 0.169). The accuracy rate did not differ between low_1_ and low_2_ (*t*_1_(316.65) = 1.45, *p* = 0.149; *t*_2_(77.44) = 1.33, *p* = 0.186). Again, we observed an interaction between polarity and truth-value (*F*_1_(2, 78) = 31.24, *p* < 0.001, η_G_^2^ = 0.06; *F*_2_(1, 114) = 45.87, *p* < 0.001, η_G_^2^ = 0.14) which reflects the relative difficulty to correctly verify true negated sentences. Predictability did not influence this interaction (*F*_1_(2, 78) = 1.63, *p* = 0.207, ε = 0.86, η_G_^2^ = 0.01; *F*_2_(2, 114) = 2.20, *p* = 0.115, η_G_^2^ = 0.02). Separate analyses for the three levels of predictability confirmed the observed overall truth-value-by-polarity interaction in every subgroup of predictability (high: *F*_1_(1, 39) = 24.49, *p* < 0.001, η_G_^2^ = 0.14; *F*_2_(1, 38) = 45.98, *p* < 0.001, η_G_^2^ = 0.38; low_1_: *F*_1_(1, 39) = 8.72, *p* = 0.005, η_G_^2^ = 0.04; *F*_2_(1, 38) = 11.86, *p* = 0.001, η_G_^2^ = 0.10; low_2_: *F*_1_(1, 39) = 5.06, *p* = 0.030, η_G_^2^ = 0.03; *F*_2_(1, 38) = 6.01, *p* = 0.019, η_G_^2^ = 0.06). For the high-predictable subgroup true negated statements are harder to correctly classify than false negated statements (*t*_1_(39) = − 5.18, *p* < 0.001; *t*_2_(19) = -6.76, *p* < 0.001). The opposite is the case for affirmative sentences (*t*_1_(39) = 2.38, *p* = 0.022; *t*_2_(19) = 2.82, *p* = 0.011). The pattern of the advantage of false negatives over true negatives also showed in the low-predictable subgroups (low_1_: *t*_1_(39) = 3.79, *p* < 0.001, *t*_2_(19) = 5.15, *p* < 0.001; low_2_: *t*_1_(39) = 2.45, *p* = 0.019, *t*_2_(19) = 2.50, *p* = 0.021). However, true affirmatives were not easier to classify than false affirmatives (low_*1*_: *t*_1_(39) = − 1.23, *p* = 0.225, *t*_2_(19) = − 1.06, *p* = 0.301; low_2_: both *t*s < 1). See Fig. 2 for percentage of correctly verified true and false sentences in the different conditions of polarity and predictability.

As was already mentioned in the description of the materials (pretest), the three levels of predictability were not fully matched with respect to the frequency and the length of the adjectives. In order to shed more light onto the relevance of these lexical variables to our findings, we conducted two additional analyses. First, we created a subgroup of our materials for which the three levels of predictability did not differ with respect to frequency (high vs. low_1_: *t* < 1; high vs. low_2_: *t*(84.95) = − 1.12, *p* = 0.266; low_1_ vs. low_2_: *t*(133.98) = − 1.48, *p* = 0.141). Second, we created a subgroup of our materials for which the three levels of predictability did not differ with respect to length (high vs. low_1_: *t*(117.54) = − 1.43, *p* = 0.156; high vs. low_2_: *t*(104.28) = − 1.67, *p* = 0.098; low_1_ vs. low_2_: *t* < 1). Both subgroups of materials were created by taking out three trials in each of the overall 12 conditions. For the analyses with items as random factors we would need to take out 36 items in total, because each of them would have missing values in at least one of the conditions. We therefore decided to just run analyses with participants as random factor for which it suffices to exclude 36 individual trials. These two analyses will be reported in what follows.

The Huynh–Feldt corrected 2 × 3 × 2 ANOVA for the subgroup controlled for frequency with participants as random factors revealed a main effect for polarity (*F*(1, 39) = 137.93, *p* < 0.001, η_G_^2^ = 0.10), predictability (*F*(2, 78) = 16.67, *p* < 0.001, η_G_^2^ = 0.01), as well as truth-value (*F*(1, 39) = 9.06, *p* = 0.004, η_G_^2^ = 0.004). Again, there was a significant interaction between polarity and truth-value (*F*(1, 39) = 12.44, *p* = 0.001, η_G_^2^ = 0.01). This interaction was modulated by predictability (*F*(2, 78) = 5.88, *p* = 0.004, η_G_^2^ = 0.005). Separate analyses for the three levels of predictability revealed the truth-value-by-polarity interaction in responses to high-predictable sentences (*F*(1, 39) = 17.67, *p* < 0.001, η_G_^2^ = 0.03), with significantly faster responses to true compared to false affirmatives (*t*(39) = − 5.67, *p* < 0.001) and a tendency for slower responses to true compared to false negatives (*t*(39) = 1.81, *p* = 0.078). There was no interaction of truth-value and polarity in the low_1_ category (*F* < 1), but only a main effect for polarity (*F*(1, 39) = 45.48, *p* < 0.001, η_G_^2^ = 0.08), reflecting faster responses to affirmative sentences, and a main effect for truth-value (*F*(1, 39) = 6.77, *p* = 0.013, η_G_^2^ = 0.01), reflecting faster response to true sentences. Again, no truth-value-by-polarity interaction showed for the low_2_ category (*F*(1, 39) = 2.34, *p* = 0.134, η_G_^2^ = 0.003). There was no main effect of truth-value (*F* < 1) but of polarity (*F*(1,39) = 72.78, *p* < 0.001, η_G_^2^ = 0.09), reflecting faster responses to affirmative compared to negative sentences.

The Huynh–Feldt corrected 2 × 3 × 2 ANOVA for the subgroup of items controlled for length with participants as random factors showed a main effect for polarity (*F*(1, 39) = 135.06, *p* < 0.001, η_G_^2^ = 0.09), predictability (*F*(2, 78) = 10.82, *p* < 0.001, η_G_^2^ = 0.01), as well as for truth-value (*F*(1, 39) = 5.22, *p* = 0.028, η_G_^2^ = 0.003). We found the truth-value-by-polarity interaction (*F*(1, 39) = 13.26, *p* < 0.001, η_G_^2^ = 0.01). There was no effect of predictability on this interaction (*F*(2, 78) = 2.42, *p* = 0.096, η_G_^2^ = 0.002). Separate analyses for the three levels of predictability revealed the truth-value-by-polarity interaction in responses to high-predictable sentences (*F*(1, 39) = 14.08, *p* < 0.001, η_G_^2^ = 0.02), with significantly faster responses to true compared to false affirmatives (*t*(39) = − 5.20, *p* < 0.001) and no differences in response times for true compared to false negatives (*t*(39) = 1.35, *p* = 0.183). This interaction was also visible in the low_1_ category (*F*(1, 39) = 5.93, *p* = 0.019, η_G_^2^ = 0.01), reflecting faster responses to true compared to false affirmatives (*t*(39) = − 2.85, *p* = 0.01), but no difference between true and false negatives (*t* < 1). There was no interaction in the low_2_ category (*F*(1, 39) = 1.74, *p* = 0.194, η_G_^2^ = 0.002), but only a main effect for polarity (*F*(1, 39) = 82.67, *p* < 0.001, η_G_^2^ = 0.09), reflecting faster responses to affirmative compared to negative sentences. There was no difference between response to true and false sentences (*F* < 1).

## Discussion

The aim of the present study was to investigate different explanations for the often-observed interaction between truth-value and polarity, reflecting a relative ease of processing false negative sentences compared to true negative sentences. The recent literature suggests two relevant factors, namely, lexical associations and predictability. To gain more information with respect to these two factors, we examined the processing of true or false affirmative and negative sentences in contexts controlled for lexical associations with varying predictability. If lexical associations are responsible for the truth-value-by-polarity interaction that was observed in recent studies, then we should have found only two main effects, one of polarity, reflecting an advantage of affirmative over negative sentences, and one of truth-value, reflecting an advantage of true over false sentences. The reason is that lexical associations—which otherwise might result in a facilitation of the processing of the false negative sentences—are ruled out as an explanation in the current study (as in the studies by Clark & Chase, 1972; Carpenter & Just, 1975). All of the analyses (with the complete data set, the data sets controlled for length and frequency and the data set without potentially lexically associated red and green crosses, and the data set containing no arrow items) showed not only main effects of polarity and truth-value, but a significant interaction of these two factors. This rules out that long-term lexical associations are responsible for the observed interaction.

In contrast, if predictability is the key factor for the truth-value-by-polarity interaction in the sense that missing predictability of the target words in true negatives makes them particularly hard to process, then we should have observed a truth-value-by-polarity interaction in particular in the low-predictable conditions in the current experiment, in alignment with Nieuwland (2016). We found that predictability had an influence on the truth-value-by-polarity interaction, which was especially pronounced in the data set controlled for frequency. Contrary to our expectations, the interaction was clearly visible in the high-predictable condition but not in the low-predictable conditions (in the data set without arrow items, the interaction was visible in the high and the low-predictable conditions. One might argue that due to task repetition in a rather restricted environment, our low-predictable conditions might have become highly predictable too. After a while, participants might notice that sometimes the sentences refer to the dimension that is less expected from the perspective of the participants. However, the fact that responses were faster in the high-predictable conditions (also in the analyses controlled for lexical variables) speaks against this idea. Also, even if this were the case, our main prediction would stay the same. In case all conditions were highly predictable due to task repetition, we should not have observed polarity-by-truth-value interactions in any of the conditions (provided that predictability plays the role it is assumed to play according to previous accounts).

Although the present results were rather clear-cut, both predictions (the ones based on lexical association and the one based on predictability) were not borne out by the data. Rather, what we observed was a truth-value-by-polarity interaction with relatively fast responses in the false negative condition, which if at all was more pronounced in the high- rather than the low-predictable conditions.

What can be concluded from these results? In our view, we can conclude that the truth-value by polarity interaction is not in all cases dependent on lexical associations being present in the true affirmative and the false negative conditions (in alignment with the early verification studies), nor in all cases dependent on low predictability of the target words in the true negative conditions. If so, we should not have observed an interaction in the present study, especially not in the high-predictable conditions. However, this does not rule out that the observed interaction may have different causes in different paradigms. In principle, it is possible that in paradigms with verification tasks measuring a dependent variable that picks up on rather late processes, a truth-value by polarity interaction emerges which reflects the relative difficulty of arriving at the final truth-value of the sentences, as suggested by Clark and Chase (1972) and Carpenter and Just (1975). In contrast, in paradigms measuring a dependent variable that picks up on comprehension processes proper, such as ERP paradigms looking at differences in N400 amplitudes, lexical associations or low predictability may lead to the observed truth-value-by-polarity interaction effect. In other words, the often observed interaction would not reflect one homogenous phenomenon but rather have different sources in different paradigms. If so, it remains to be explained why a truth-value-by-polarity interaction was observed in the study by Kaup et al. (2005) in which participants were neither required to verify the sentences, nor were there any differences between the conditions with respect to lexical associations. One possibility is that participants spontaneously verify sentences as part of comprehension if the presented information allows them to. Indeed, this assumption fits well with research on monitoring during language comprehension suggesting that verification processes are an integral part of comprehension rather than an optional post-comprehension process (e.g., Herbert & Kißler, 2014; Isberner & Richter, 2013; Richter, 2015; Singer, 2006, 2013).

Up to now, we excluded lexical associations as a key factor for explaining the results observed in our current study. The reason is that there should be no lexical associations between the geometrical shapes (e.g., square, circle, triangle) and the properties (e.g., blue, dotted, large, bright) employed in our materials. This, in our view, certainly holds for the kind of long-term lexical associations that stem from our linguistic and non-linguistic experiences in the past. Admittedly, it seems that at least some people associate certain shapes with certain colors. This is shown when people are prompted to pick a color for a white geometrical form with black outlines (Albertazzi et al., 2013). However, we consider it highly unlikely that we systematically selected these associations in particular conditions in our experiment. But what about short-lived lexical associations that might be spontaneously built when viewing the visual displays in our experiment? In other words, we consider it conceivable that participants internally verbalize the visual displays and that this might give rise to short-lived lexical associations. For instance, when viewing a display with a large blue circle and a large green square, a participant might internally verbalize the image as “There is a blue circle and a green square*”* leading to associations between “blue” and “circle” and between “square” and “green”. Thus, when reading a true affirmative or a false negative sentence (e.g., “The square is (not) green”), these lexical associations may facilitate processing compared to conditions in which a false affirmative or true negative sentence is presented (e.g., “The square is (not) blue”) for which such spontaneous associations are not present. We are not aware of any studies explicitly investigating the question whether, and if so, under which conditions such short-lived spontaneous lexical associations are at play. However, in principle, such an explanation would fit well with the many studies on the relationship between language and thought, showing that people tend to verbalize non-linguistic stimuli even in tasks where this is unnecessary and even detrimental (e.g., Lupyan et al., 2012; Nakabayashi et al., 2012; Souza & Skóra, 2017, for an overview see Kaup & Ulrich, 2017). Future studies are necessary to shed light on this potential explanation. One possibility is the use of articulatory suppression to prevent participants from internally verbalizing the images. Another interesting option would be to investigate whether these potential short-term associations also lead to a truth-value-by-polarity interaction in ERP studies investigating N400 amplitudes even if participants are not asked to verify the sentences.

Finally, before closing, we would like to point out a different, rather speculative explanation of our results. Specifically, there may be pragmatic reasons why false negated statements are relatively easy to process compared to true negated statements. Negation is typically used when a state of affairs deviates from the norm (e.g., Valle Arroyo, 1982; Wason, 1965) or when the speaker wishes to communicate that something differs from our expectations (e.g., Glenberg et al., 1999; Nordmeyer & Frank, 2014). When this is taken at face value, then false negated statements might be particularly felicitous in a pragmatic sense, because here negation is exactly used in this way, namely, to convey something that deviates from the norm or is unexpected. Thus, a sentence such as “Zebras are not stripy” could be considered to be pragmatically informative by describing a state of the world that differs from our knowledge of how the world normally is. In contrast, true negative sentences such as “Zebras are not dotted” might be pragmatically uninformative as here the negation is used to state a common fact. Pragmatic informativeness, in this case, is about using the negation for conveying unexpected new information, which might bring its own added value independently of truth-value. It might seem contra-intuitive that falsity should be associated with processing ease rather than processing difficulty. However, in our view, this assumption would fit well with the rather general assumptions made in the literature on the pragmatics of negation (e.g., Givón, 1978; Horn, 1985), and probably constituted the basis for the well-known statement: *In real life negatives are false!* (Wason, 1972).

## Conclusions

The results of the present study show that the often-observed truth-value-by-polarity interaction, reflecting a relative processing ease for false negative sentences, is neither dependent on long-term lexical associations nor on presenting negation within a low-predictable context. Future studies are needed to determine the role of short-term lexical associations that might come about through spontaneous internal verbalization processes when participants inspect visual displays in a sentence-picture verification paradigm. In addition, more research is needed investigating factors related to informativeness in negative sentences.

## Data Availability

We will save the data together with the publication in the online database The Mind Research Repository.

## Appendix 1

Examples for visual worlds and sentences in the different conditions of predictability.

## Appendix 2

### Analysis without possibly lexically associated items (red and green cross)

We excluded 12 items with a green or red cross and conducted the two Huynh–Feldt corrected 2 × 2 × 3 ANOVAs with the factors polarity (affirmative, negated), truth-value (true, false) and predictability (high, low_1_, low_2_) on response times.One of these ANOVAs treated participants as random factor, the other treated items as random factor. Without these 12 items, responses were still slower for negated compared to affirmative statements (*F*_1_(1, 39) = 126.96, *p* < 0.001, η_G_^2^ = .09; *F*_2_(1, 102) = 163.88, *p* < 0.001, η_G_^2^ = .40). Responses to false statements now still tended to be slower compared to true statements (*F*_1_(1, 39) = 3.57, *p* = 0.066, η_G_^2^ = .002; *F*_2_(1, 102) = 3.04, *p* = 0.084, η_G_^2^ = .02). Like in the complete data set, response times differed for the three levels of predictability (*F*_1_(2, 78) = 21.19, *p* < 0.001, η_G_^2^ = .01; *F*_2_(2, 102) = 10.18,* p* < 0.001, η_G_^2^ = .08), reflecting the advantage of high-predictable statements compared to statements in the category low_1_ (*t*_1_(315.73) = − 2.06, *p* = 0.040; *t*_2_(64.79) = − 2.48, *p* = 0.015) and low_2_ (*t*_1_(317.86) = − 1.95, *p* = 0.052; *t*_2_(73.59) = − 2.43, *p* = 0.017), and no difference between the two low categories (both *t*s < 1). Predictability did not interact with truth-value (*F*_1_ < 1, ε = 0.84; *F*_2_ < 1) nor with polarity (both *F*s < 1). The interaction between polarity and truth-value was again significant (*F*_1_(1, 39) = 12.36*, p* = 0.001, η_G_^2^ = .01; *F*_2_(1, 102) = 9.76*, p* = 0.002, η_G_^2^ = .05). There was still no influence of predictability on the truth-value-by-polarity interaction (*F*_1_(2, 78) = 1.76,* p* = 0.178, η_G_^2^ = .002; *F*_*2*_(2, 102) = 1.70,* p* = 0.187, η_G_^2^ = .02).

Separate analyses for the three levels of predictability revealed the interaction between truth-value and polarity only in high-predictable combinations (*F*_1_(1, 39) = 11.25, *p* = 0.002, η_G_^2^ = .02; *F*_2_(1, 36) = 12.13,* p* = 0.001, η_G_^2^ = .18), with faster responses to true affirmatives compared to false affirmatives (*t*_1_(39) = − 5.27, *p* < 0.001; *t*_2_(18) = − 4.02, *p* < 0.001), and no significant differences in response times for true negatives compared to false negatives (*t*_1_(39) = 1.16, *p* = 0.252; *t*_2_(18) = 1.14, *p* = 0.270). Again, we did not see the interaction between truth-value and polarity in low-predictable combinations, at least not in both the by-participant as well as the by-item analyses (low_1_: *F*_1_(1, 39) = 4.47,* p* = 0.041, η_G_^2^ = .005; *F*_2_ < 1; low_2_: *F*_1_(1, 39) = 1.31,* p* = 0.260, η_G_^2^ = .003; *F*_2_(1, 36) = 1.06,* p* = 0.309, η_G_^2^ = .02). In the low_1_ condition, negative statements took longer to verify compared to affirmative statements (*F*_1_(1, 39) = 54.20, *p* < 0.001, η_G_^2^ = .07; *F*_2_(1, 30) = 49.87, *p* < 0.001, η_G_^2^ = .41). The tendency of false statements taking longer to verify than true statements was no longer visible in the reduced data set (both *F*s < 1). The relative ease to classify affirmative sentences also showed in the low_2_ condition (*F*_1_(1, 39) = 52.47, *p* < 0.001, η_G_^2^ = .09; *F*_2_(1, 36) = 40.12, *p* < 0.001, η_G_^2^ = .35). Like in the complete dataset, there was no difference in response times between true and false sentences (both *F*s < 1).

### Analysis without items containing arrows

We excluded 31 items in which one form was an arrow and conducted the two Huynh-Feldt corrected 2 × 2 × 3 ANOVAs with the factors polarity (affirmative, negated), truth-value (true, false) and predictability (high, low_1_, low_2_) on response times.One of these ANOVAs treated participants as random factor, the other treated items as random factor. Response times were slower for negated compared to affirmative statements (*F*_1_(1, 39) = 203.87, *p* < 0.001, η_G_^2^ = .10; *F*_2_(1, 83) = 109.57, *p* < 0.001, η_G_^2^ = .40) and for false compared to true statements (*F*_1_ (1, 39) = 3.85, *p* = 0.057, η_G_^2^ = .003; *F*_2_(1, 83) = 4.22, *p* = 0.043, η_G_^2^ = .02). There was also a main effect of predictability (*F*_1_(2, 78) = 11.51, *p* < 0.001, η_G_^2^ = .01; *F*_2_(2, 83) = 3.58, *p* = 0.032, η_G_^2^ = .04), reflecting an advantage of the high predictable condition over the low_2_ condition (*t*_1_(317.04) = − 1.74, *p* = 0.083, *t*_2_(53.91) = − 2.08, *p* = 0.043) but not over the low_1_ condition (both *t*s < 1). Predictability did not interact with polarity (*F*_1_(2, 78) = 2.92, *p* = 0.060, η_G_^2^ = .002; *F*_2_ < 1) nor with truth-value (*F*_1_(2, 78) = 1.89, *p* = 0.164, ε = .86, η_G_^2^ = .001; *F*_2_ < 1). The interaction between polarity and truth-value was significant (*F*_1_(1, 39) = 24.02*, p *< 0.001, η_G_^2^ = .01; *F*_2_(1, 83) = 14.38*, p *< 0.001, η_G_^2^ = .08). There was no influence of predictability on the truth-value-by-polarity interaction (both *F*s < 1).

Separate analyses for the three levels of predictability revealed the truth-value-by-polarity interaction in the high-predictable condition (*F*_1_(1, 39) = 14.45, *p* < 0.001, η_G_^2^ = .02; *F*_2_(1, 25) = 7.55, *p* = 0.012, η_G_^2^ = .16) with faster responses to true compared to false affirmatives (*t*_1_(39) = − 4.26, *p* < 0.001; *t*_*2*_(14) = − 3.35,* p* = 0.005) and no significant difference between true and false negatives (*t*_1_(1, 39) = 1.67, *p* = 0.103, *t*_2_(11) = 0.94, *p* = 0.368). This time, the interaction was also visible in the low-predictable conditions (low_1_: *F*_1_(1, 39) = 9.87, *p* = 0.003, η_G_^2^ = .01, *F*_2_(1, 31) = 1.56,* p* = 0.220, η_G_^2^ = .03; low_2_: *F*_1_(1, 39) = 5.63, *p* = 0.023, η_G_^2^ = .01, *F*_2_(1, 27) = 7.93, *p* = 0.009, η_G_^2^ = .09), again, reflecting faster responses to true than false affirmatives (low_1_:* t*_1_(39) = − 4.36, *p* < 0.001, *t*_2_(16) = − 3.01, *p* = 0.008; low_2_: *t*_1_(39) = − 2.10, *p* = .042, *t*_2_(13) = − 2.34, *p* = 0.036) and no difference between true and false negated statements (low_1_: *t*_1_(39) = -0.30, *p* = 0.765, *t*_2_(15) = −0.22, *p* = 0.832; low_2_: *t*_1_(39) = 1.55, *p* = 0.130, *t*_2_(14) = 1.68, *p* = 0.116).

### Analysis with data from only the first half of the experiment

To see, whether the truth-value-by-polarity interaction was not only due to a strategy developed by participants in the course of the experiment, we ran the analyses with data from the first half of the experiment (with the exclusion of one participant in the by-participants analysis, due to missing data in one condition). The overall analysis again revealed a main effect for polarity (*F*_1_(1, 38) = 80.09, *p* < 0.001, η_G_^2^ = .09; *F*_2_(1, 114) = 82.08, *p* < 0.001, η_G_^2^ = .26), reflecting faster responses to affirmative compared to negative statements. A main effect of predictability already emerged in the first experimental half (*F*_1_(2, 76) = 6.75, *p* = 0.002, η_G_^2^ = .01; *F*_2_(2, 114) = 5.72, *p* = 0.004, η_G_^2^ = .05), reflecting an advantage of high-predictable statements over both low_1_- and low_2_-predictable statements, at least in the by-items analysis (low_1_: *t*_1_(309.88) = − 1.46, *p* = 0.144, *t*_2_(77.79) = − 2.02, *p* = 0.046; low_2_: *t*_1_(309.44) = − 1.64, *p* = 0.102, *t*_*2*_(77.99) = − 2.75, *p* = 0.016) and no difference between the two low conditions (both *t*s < 1). There was no main effect of truth-value in the first half of the experiment (*F*_1_(1, 38) = 2.95, *p* = 0.09, η_G_^2^ = .003; *F*_2_(1, 114) = 2.67, *p* = 0.105, η_G_^2^ = .01). Crucially, there was a significant interaction between polarity and truth-value (*F*_1_ (1, 38) = 10.21, *p* = 0.003, η_G_^2^ = .01; *F*_2_(1, 114) = 7.11, *p* = 0.008, η_G_^2^ = .03). Predictability neither interacted with polarity (both *F*s < 1) nor with truth-value (both *F*s < 1) nor did it influence the interaction between polarity and truth-value (*F*_1_ (2, 76) = 2.03, *p* = 0.138, η_G_^2^ = .001; *F*_2_ (2, 114) = 1.39, *p* = 0.253, η_G_^2^ = .01).

Like with the complete data set, the three separate analyses for the different levels of predictability showed the interaction in the high predictable condition (*F*_1_(1, 38) = 9.17, *p* = 0.004, η_G_^2^ = .02; *F*_2_ (1, 38) = 8.90, *p* = 0.005, η_G_^2^ = .11) with faster response times for true compared to false affirmatives (*t*_1_(38) = − 3.97, *p* < 0.001; *t*_2_(19) = − 4.05, *p* < 0.001), and no difference between true and false negatives (*t*_1_(38) = 1.06, *p* = 0.296; *t*_*2*_ < 1). Again, we did not see the interaction between truth-value and polarity in low-predictable combinations, at least not in both the by-participant as well as the by-item analyses (low_1_: *F*_1_(1, 38) = 6.29,* p* = 0.002, η_G_^2^ = .01; *F*_2_(1, 38) = 1.13,* p* = 0.295, η_G_^2^ = .01; low_2_: both *F*s < 1). The main effect of polarity reflects the advantage of affirmative over negative statements in both low-categories (low_1_:* F*_1_(1, 38) = 42.99, *p* < 0.001, η_G_^2^ =.10, *F*_2_(1, 38) = 26.49,* p* < 0.001, η_G_^2^ = .25; low_2_: *F*_1_(1, 38) = 34.44, *p* < 0.001, η_G_^2^ = .07, *F*_2_(1, 38) = 21.73,* p *< 0.001, η_G_^2^ = .23). There was no main effect of truth-value (all *Fs* < 1).

## Footnotes

1 Our material included 12 items with a solid red or green cross. A red or green cross is a typical symbol to denote a hospital or a pharmacy. We ran our analyses without these items and found the same interactions as with the whole data set. Also, to see whether the truth-value-by-polarity interaction was not only due to a strategy developed by participants in the course of the experiment, we ran the analyses with data from the first half of the experiment (with the exclusion of one participant in the by-participants analysis, due to missing data in one condition). The overall analysis, as well as the separate analyses for each predictability condition showed essentially the same result pattern as the analyses with both experimental halves. See the Appendix 2 for both analyses. We thank an anonymous reviewer for suggesting these additional analyses.
